# Pre-clinical models to study abnormal uterine bleeding (AUB)

**DOI:** 10.1016/j.ebiom.2022.104238

**Published:** 2022-09-05

**Authors:** Aleksandra O. Tsolova, Rocío Martínez Aguilar, Jacqueline A. Maybin, Hilary O.D. Critchley

**Affiliations:** MRC Centre for Reproductive Health, University of Edinburgh, Edinburgh, UK

**Keywords:** Abnormal uterine bleeding (AUB), Endometrium, Pre-clinical models, Epithelial organoids, Endometrial co-cultures, Adenomyosis, Leiomyoma (uterine fibroids)

## Abstract

Abnormal Uterine Bleeding (AUB) is a common debilitating condition that significantly reduces quality of life of women across the reproductive age span. AUB creates significant morbidity, medical, social, and economic problems for women, their families, workplace, and health services. Despite the profoundly negative effects of AUB on public health, advancement in understanding the pathophysiology of AUB and the discovery of novel effective therapies is slow due to lack of reliable pre-clinical models.

This review discusses currently available laboratory-based pre-clinical scientific models and how they are used to study AUB. Human and animal *in vitro, ex vivo*, and *in vivo* models will be described along with advantages and limitations of each method.

## Abnormal uterine bleeding (AUB) definition, diagnosis, and need for pre-clinical models

Endometrial function through the menstrual, proliferative, and secretory phases is tightly regulated by hormonal endocrine signalling. Circulating ovarian sex steroids oestrogen and progesterone are crucial for this regulation. Oestrogen stimulates endometrial cell proliferation and regeneration following menstruation; progesterone opposes oestrogen-stimulated proliferation and stimulates cell differentiation in preparation for implantation. If pregnancy does not occur, progesterone is withdrawn, and this triggers menstruation as the new menstrual cycle begins.^1^ Dysregulation of these processes may lead to abnormalities in endometrial breakdown, bleeding, repair, and regeneration.

Normal uterine bleeding is defined using four parameters: frequency, duration, volume, and regularity. A typical menstrual cycle (i) has a frequency of 24–38 days, (ii) a regularity (cycle to cycle variation) of 2–20 days, (iii) has a duration of 4.5–8 days, and (iv) has a volume of 5–80 millilitres.

Abnormal Uterine Bleeding (AUB) is a highly prevalent symptom of an underlying condition/s experienced by one in three women of reproductive age. AUB, which includes heavy menstrual bleeding (HMB), causes significant morbidity and affects every aspect of the sufferer's life. A clear definition, description of the causes, and signs of AUB has been established, which “*should assist in providing a solid basis for the standardisation of international research and clinical manuscripts addressing the diagnosis, pathogenesis, and management of AUB*”.^2^^,^^3^ The International Federation of Obstetrics and Gynaecology (FIGO) Menstrual Disorders Committee (MDC) published the two FIGO systems (terminology; System 1 and classification; System 2) in 2011 with revisions in 2018.^4^

System 1 defines AUB as a symptom presenting values of menstrual bleeding frequency, duration, volume, and regularity outside of the normal range described above.^2^ Acute AUB is an episode of heavy menstrual bleeding (HMB) that requires immediate intervention. Chronic AUB is AUB present for most of the past 6 months that does not require urgent medical attention.^3^

**Figure 1:**
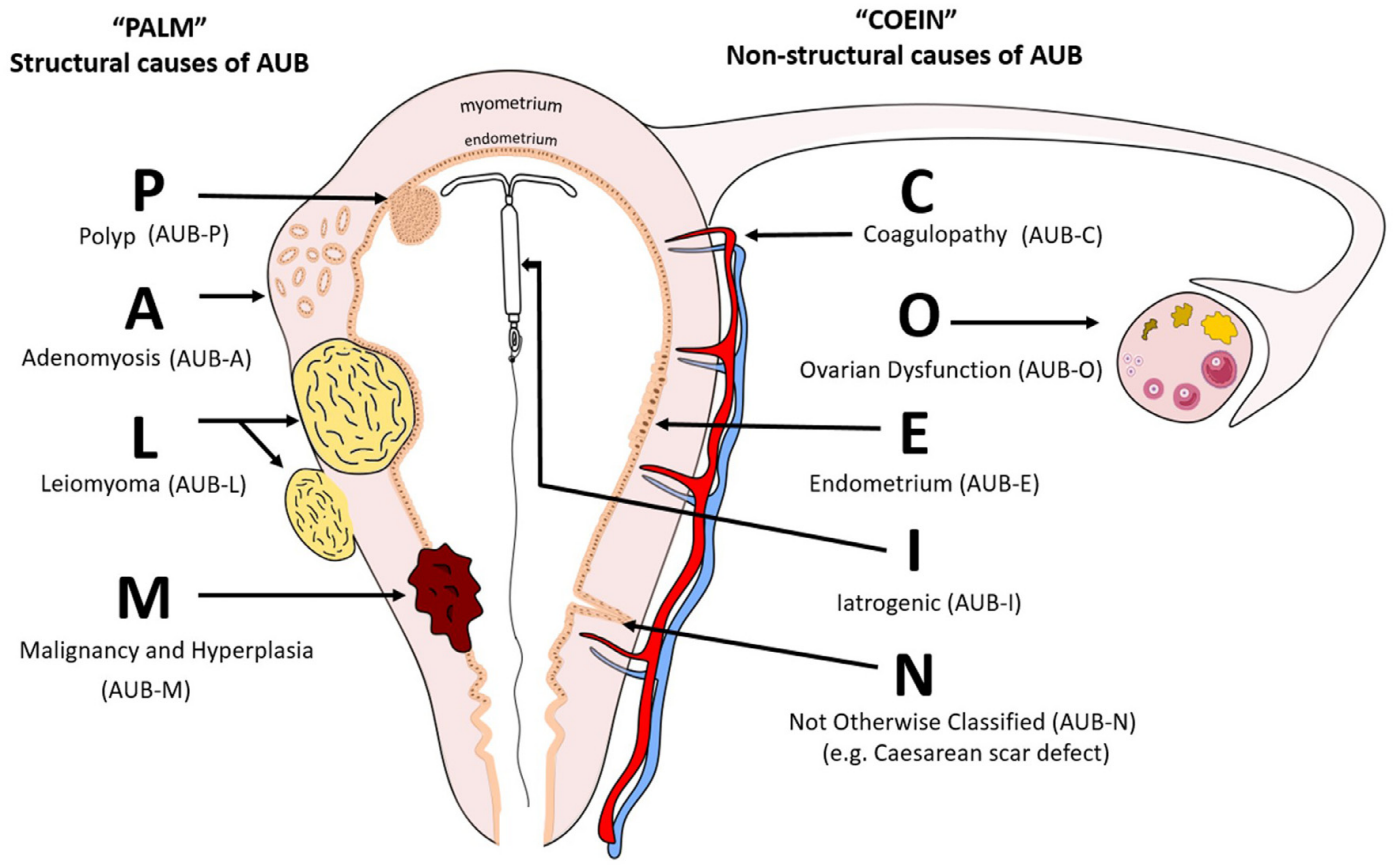
**FIGO classification of abnormal uterine bleeding (AUB) – structural (PALM) and non-structural (COEIN) causes (4)**.

FIGO system 2 classifies AUB using the acronym PALM-COEIN [*pahm-koin*] describing two groups of AUB aetiologies – “**PALM**” describes the structural, and “**COEIN**” - the non-structural causes (Figure 1).

Abnormal uterine bleeding can be caused by none, one, or multiple PALM-COEIN aetiologies. Some may be present but asymptomatic (AUB-L, see Figure 1), and others can only be diagnosed by exclusion of other identifiable factors (such as underlying endometrial anomaly, AUB-E).^4^ This classification system is essential for scientific and clinical progress in this area. Non-structural causes such as AUB-E require delineation of mechanism of action for treatment improvement, whereas structural causes require delineation of endometrial impact as a cause (or lack) of AUB. Only by gaining understanding of the above, can we then address the complexity of the presence of more than one cause of AUB in a single patient.

Advancement in our understanding of endometrial pathophysiology has been possible through use of modern cellular and molecular techniques, as well as animal models. This has facilitated investigation of therapeutic targets and new treatment options in the wider goal of improving precision for patient management.^1^

## Definition, purpose, and validity of a pre-clinical model

Broadly, pre-clinical modelling is any experimental preparation in a laboratory setting which enables an in-depth investigation of a complex biological phenomenon.

Essential to the development of a pre-clinical model is the consideration of the model's intended purpose. This will establish validation requirements of the model. In the context of studying human menstrual disorders, i.e., AUB and endometrial function, a pre-clinical model must present the same normal physiological response to sex steroid hormones as is observed physiologically*.* To study endometrial cell-cell interactions, the model needs construction with endometrium-derived cells with preserved phenotype when extracted from their natural microenvironment. In other cases, the model requires the use of undifferentiated progenitor cells, and their phenotype must be induced *in vitro* to reflect the tissue of origin. *In vitro* models have advanced to offer sophisticated approaches to mimic normal endometrial physiology in 3D environments. This makes them a preferred method when studying cell interactions and function as their scientific potential lies in the deconvolution of the system and ease of manipulation. If the purpose of the model, however, is to explore physiological functions or systemic interactions between organs, whole organism models such as mouse or non-human primates may be better suited or complement the *in vitro* systems.

## Uterine composition as a basis for designing a pre-clinical model to study AUB

The first step in designing a pre-clinical model to study AUB is an understanding of the individual components of the uterus and the endometrium.

**Figure 2:**
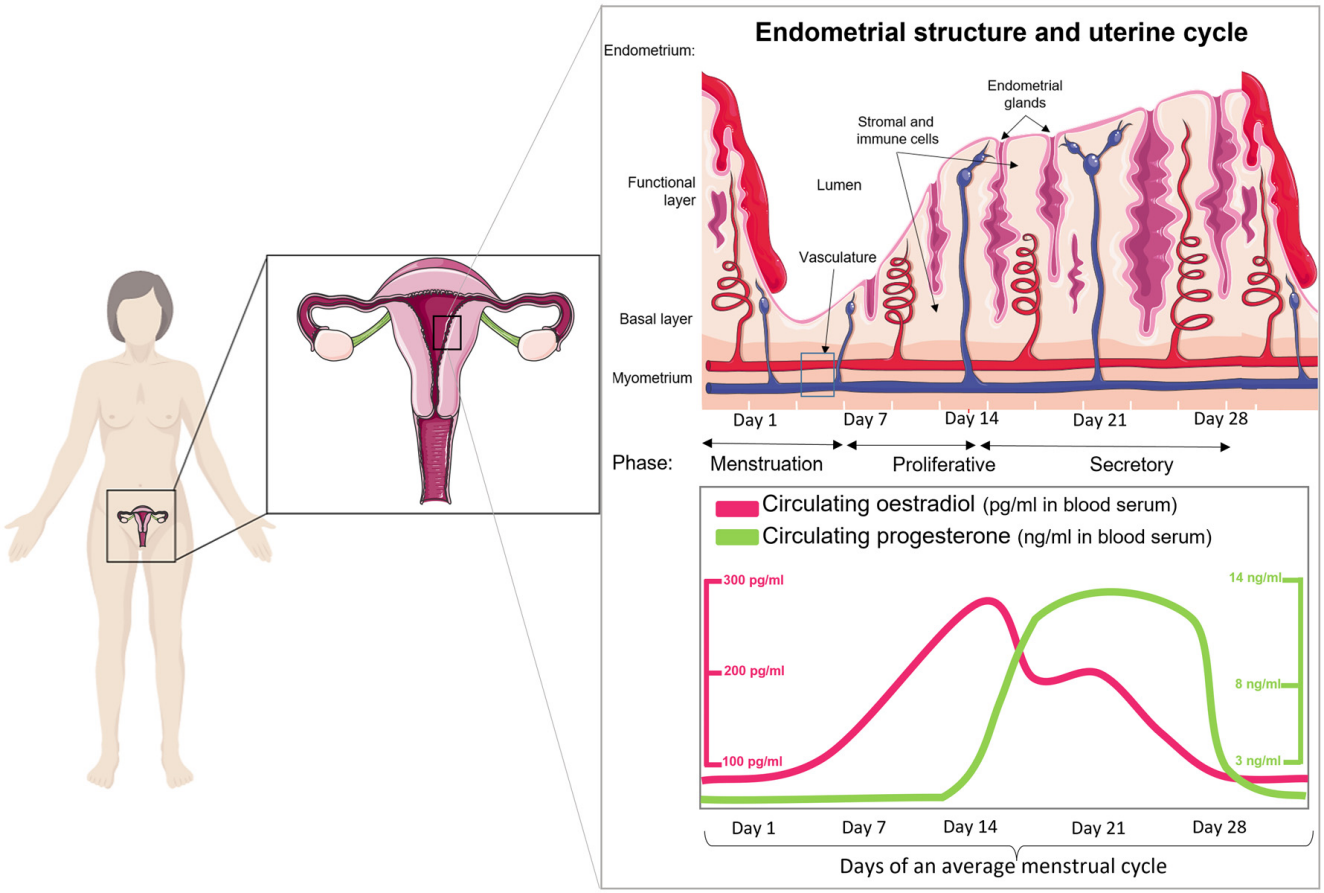
**The menstrual cycle comprises three functionally distinct phases – proliferative, secretory, and menstrual, that are regulated by sex steroid hormones – oestrogen and progesterone**.

The uterus comprises the outer serosa, myometrium, and the endometrium. The myometrium is a smooth muscle cell multilayer that undergoes contraction during labour and is the site of the structural causes of AUB, adenomyosis and fibroids (also known as leiomyomata or leiomyomas).^4^ The endometrium is composed of two layers: the basal layer, containing progenitor cells, and a functional layer, which serves as the site for embryo implantation if pregnancy occurs (Figure 2, top). The function of both layers is regulated by circulating oestrogen and progesterone (Figure 2, bottom). In the absence of pregnancy, the functional layer of the endometrium sheds during menstruation and is then rapidly repaired through a complex sequence of events involving inflammation, angiogenesis, and tissue remodelling.^1^

The endometrium is a multicellular tissue comprising stromal, epithelial, endothelial, and immune cells including uterine natural killer cells (uNK), neutrophils and macrophages.^1^^,^^5^ Modern cellular techniques employ these endometrial cells for the development of several pre-clinical models to delineate endometrial pathophysiology.

## Current pre-clinical models to study human endometrium and AUB

### Human *in vitro* models

The most widely used *in vitro* models for the study of endometrial function include primary endometrial cell cultures (stromal and epithelial cells) and organotypic cultures of epithelial cells. These may be obtained from a human endometrial biopsy or menstrual fluid, or the non-invasive alternative of using immortalised endometrial cell lines. Endometrial cell culture models offer a unique opportunity to study functional differences between the different endometrial cell subpopulations under strictly modulated growth and hormone stimuli.

### Primary endometrium-derived stromal cells (ESCs)

Isolation and culture of primary endometrial stromal cells is a one of the biggest advancements in the efforts of delineating endometrial physiology.

**Figure 3:**
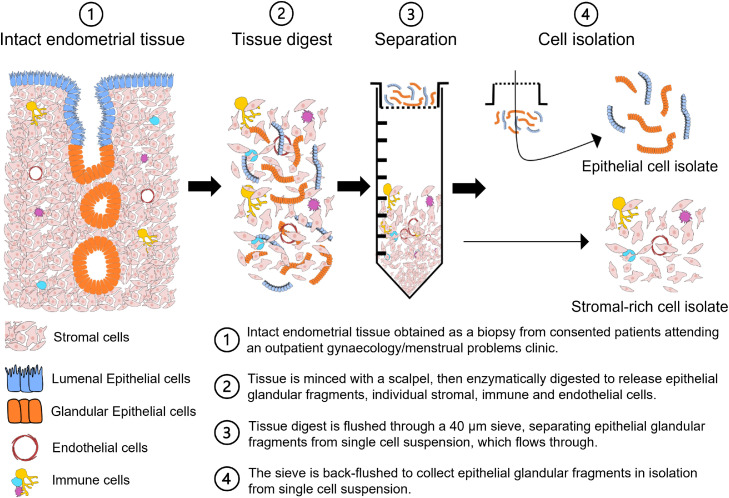
**Current standard endometrial cell isolation method.** Enzymatic digestion was performed on an endometrial biopsy from consented individuals. To separate single cell suspension, containing stromal cells, innate immune cells, and endothelial cells, from epithelial glandular fragments, the digest was flushed through a 40 µm sieve. The single cell suspension was pelleted, and epithelial glandular fragments back flushed and pelleted separately. Only stromal cells remain attached to the dish, forming a monolayer monoculture.

**Figure 4:**
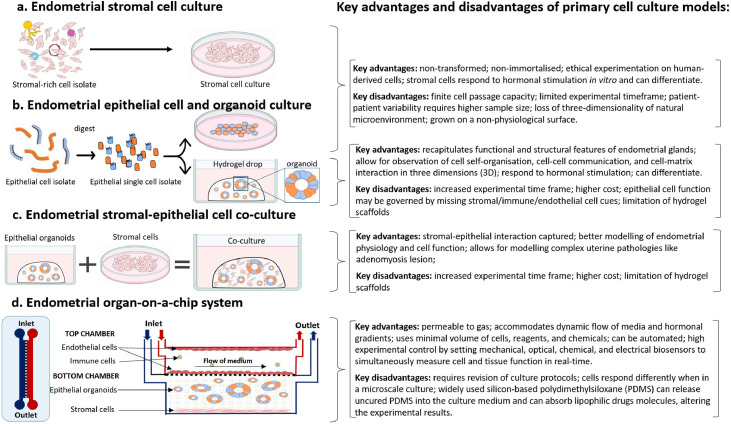
**Models of endometrial stromal and epithelial cell cultures in isolation and in a co-culture.** a) Following isolation of single cell suspension, containing stromal cells, innate immune cells, and endothelial cells, the cells were plated on a tissue culture petri dish, where only the stromal cells remain attached to the dish as they form a monolayer monoculture. b) The epithelial glandular fragments can be pelleted, re-suspended in culture medium, and plated on a tissue culture petri dish or embedded in a hydrogel droplet and cultured with a specialised medium to stimulate organoid formation.^35^^,^^36^ c) Epithelial cells are grown in a hydrogel to form organoids, while stromal cells are seeded and cultured in a Petri dish in a monolayer. When cells have reached confluence, they are lifted and combined as a co-culture within a hydrogel. d) Endometrial organ-on-a-chip system which can combine multiple endometrial cell types and create a complex endometrial microenvironment ex utero.

One of the earliest reports to describe the preparation of an endometrial stromal cell suspension was published in 1973, in which the authors used a tissue digestion medium with trypsin, collagenase, and dispase for the dissociation of rat endometrial cells from the whole tissue fragment.^6^ Soon after, the negative impact of trypsin on cell microtubules and cytoskeletal elements was demonstrated and another method was developed using manual endometrial scraping.^7^^,^^8^ The cell yield with the latter method, however, was significantly lower and the utility of the earlier described trypsin-free collagenase and dispase digestion medium has remained as the gold standard of human endometrial stromal cell isolation (Figure 3, Figure 4a).^9^^,^^10^ Over time, culture conditions have been optimised with growth factors and serum to reflect the natural microenvironment of the endometrium. Stromal cells respond to ovarian hormonal stimuli, and this has allowed for *in vitro* modelling of decidualisation.^9^ Literature on endometrial-derived stromal cells is now extensive with over 2300 publications in the last 10 years.

As primary cells, they are non-transformed and non-immortalised which enables for closer simulation of the molecular and biochemical processes to endometrial stromal cells *in vivo*. Primary ESCs are a widely used model for studying endometrial stromal-specific cell function, morphology, and differentiation.^9^^,^^11^, ^12^, ^13^, ^14^, ^15^ Understanding these processes establishes a more solid foundation for comparison with pathological cells and may elucidate therapeutic targets for AUB. For example, recent publications show their value for studying angiogenesis *in vitro*.^16^ Angiogenesis is the physiological process of vascular repair following menstruation. It is fundamental for adequate endometrial regeneration and its dysregulation may result in the symptom of AUB.^17^ This model also allows for ethical experimentation on human-derived tissue while reducing the need for animal models.

Nonetheless, utility of primary ESCs carries some limitations. Primary cells are derived from differentiated tissue biopsies and have finite cell passage capacity (reliably cultured for up to six consecutive passages) before they acquire senescent phenotype, leading to irreversible cell cycle arrest.^18^^,^^19^ This limits experimental time frames and requires constant availability of endometrial biopsies. Cells isolated from different donors may also behave differently, which requires increased sample size for each experiment. Finally, caution should be taken when obtaining results from stromal monolayer monocultures. These cells are extracted from their natural microenvironment, natural cues and cell-network communication are removed, and cells are grown on plastic or glass surface, which may alter their natural response to external influences such as hormonal treatments.

### Secondary cell lines as a model for AUB due to endometrial malignancy or hyperplasia

Endometrial cell lines differ from primary cell culture as most are derived from endometrial adenocarcinomas. Cell lines are a widely accepted model to study cellular function in malignant endometrium *in vitro*. Most well-known endometrial epithelial cell lines include Ishikawa, derived from a 39-year-old Japanese patient with a well-differentiated tumour,^20^ and HEC-1-A and HEC-1-B, derived from a 71-year-old patient with a poorly differentiated tumour.^21^ The molecular characteristics of these cell lines represent the features of grade 1 and grade 2 endometrial cancers, respectively, making them suitable to investigate cellular mechanisms underpinning AUB, including when associated with malignancy - “AUB-M”.^22^^,^^23^ Both cell lines are oestrogen and progesterone responsive, which makes them ideal candidates for studies of steroid-dependent gene expression patterns, cell signalling pathways, and receptor activity.^20^^,^^21^ In addition, they have provided insights on the importance of adiponectin,^24^ fibronectin^25^ and bone morphogenetic protein (BMPs) signalling^26^ in endometrial cancer pathogenesis, as well as oestrogen-induced angiogenesis in adenomyosis development.^27^

Human endometrial stromal cell (HESC) lines are also available. Introduction of telomerase into cultured HESCs by transfection prevents the natural chromosomal telomere shortening during mitosis, making the HESCs immortal.^28^ The telomerase transfected HESCs (THESCs) present similar morphological and functional features as the primary parent stromal cells and have been useful in studying the role of UV light application as a treatment option for endometrial hyperplasia and the therapy's effect on endometrial cell apoptosis.^28^^,^^29^ While telomerase transfection does not alter the biochemical endpoints of cell decidualisation (e.g., increased production of prolactin, insulin growth factor binding protein-1, and fibronectin), it does decrease the quantity of these proteins detected in THESCs compared to primary HESCs.^28^

Due to their immortalised phenotype, cell lines provide unlimited supply of material, making them cost effective and easy to use. They also offer highly reproducible results and bypass the ethical concerns that come with experimentation on animal and human tissue.

Like other models, however, cell lines have limitations. To be immortalised, the cells undergo genetic modification that may also alter their native function and response to stimuli. Continuous passaging may also cause genetic variation and a drift from original phenotype; hence, they cannot adequately reflect primary cell behaviour. Cell lines also carry a higher risk of cross-contamination with other cell lines or mycoplasma.^30^ The HeLa cell line, isolated from an aggressive cervical adenocarcinoma, is the most frequent contaminator of mammalian cell lines including the human endometrial epithelial cell line HES.^31^^,^^32^ Thus, working with cell lines requires continuous diligence and cell authentication that ensures cell culture purity. Although they are a powerful tool in research, cell line results should ideally be confirmed with primary cell culture experiments.^33^

### Primary endometrial gland-derived epithelial cells: a two-dimensional culture

Human endometrial epithelial cells can also be isolated from an endometrial biopsy following the same isolation procedure as primary stromal cells (Figure 3). The method was developed in 1970s and has been updated for maximum efficiency over succeeding years.^34^, ^35^, ^36^ The optimised technique uses trypsin-free digestion medium to obtain epithelial fragments and single cell suspension, which are separated using a 40 µm filter. While the stromal cell suspension flows through the filter, the epithelial glandular fragments remain on the filter until they are reverse washed. To eliminate contamination with stromal cells, the isolation technique includes a “selective attachment” step, which incubates the reverse washed cell suspension with serum-rich medium. During this step any stromal cells become attached to the petri dish, while the epithelial fragments are aspirated and plated in a separate synthetic extracellular matrix-coated cell culture plate (Figure 4b).^37^

Gland-derived endometrial epithelial cells (EECs) cultured in a two-dimensional system have been a valuable model for studying the regenerative capacity of the human endometrium. Cervelló *et. al.* reports that EEC cultures contain a unique somatic stem cell population hypothesised to be responsible for endometrial regeneration following menstruation.^38^ In addition, EECs have been invaluable for studying the cell molecular signature during the implantation window.^39^^,^^40^

Endometrial epithelial cells offer the same benefits to research as primary stromal cells but carry some major limitations. While the method incorporates techniques to limit presence of stromal cells, contamination is common and often stromal cell growth overtakes the epithelial cell cultures.^37^ In addition, two-dimensional (2D) *in vitro* EEC cultures do not reproduce the full complexity of the natural topographical environment that supports glandular function.^41^ Grown in 2D and on cell culture appropriate plastic ware, these EEC cultures degenerate after 10-15 days, making them unviable for passaging, culture propagation, and model standardisation.^34^ These cultures are also unresponsive to oestrogen and progesterone, making them unable to differentiate and incompetent for functional studies.^34^^,^^35^ Hence, new strategies were employed to create a three-dimensional (3D) culture system, which was transformative for the field – the development of epithelial organoids.

### Primary endometrial gland-derived epithelial organoids: a three-dimensional culture

Following the identification of adult stem cells in variety of adult organs, in 2009 Sato et al described the generation of a 3D intestinal culture system (organoids) that has thereafter led to the establishment of organoids from multiple other tissues.^42^ Since 2009, over 8500 papers have been published that make use of organoids, demonstrating their applicability to a wide range of studies, including of the endometrium.^43^, ^44^, ^45^

Endometrial epithelial organoids (EEOs) are self-organising epithelial 3D culture systems that retain functional and morphological features of the endometrial glands *in vitro,* while allowing flexibility for experimental manipulation.^43^^,^^44^ For organoid development, isolated EECs are embedded into an extracellular matrix such as the commercially available Matrigel®, an Engelbreth-Holm-Swarm mouse sarcoma-derived gelatinous protein mixture (Figure 4b).^43^^,^^44^ The EECs contain populations of progenitor stem cells and differentiated epithelial cells. These cells are then cultured in basal medium supplemented with a cocktail of growth and signalling factors that support organoid formation, growth, and culture expansion.^43^^,^^44^^,^^46^ Recently, Cindrova-Davies et al, using similar methods as derivation from endometrial biopsies demonstrated that menstrual flow has potential as an alternative, non-invasive source of primary sample/tissue/effluent, offering a more accessible resource to establish endometrial epithelial organoid cultures.^47^

Three-dimensional EEO cultures are widely accepted to accurately represent functional and structural features of *in vivo* endometrial glands. The 3D model allows experimental observation of cell self-organisation, cell-cell communication, and cell-matrix interaction in a physiologically relevant environment in all three dimensions that has not been possible with two-dimensional alternatives. EEOs respond to hormonal stimulation *in vitro* to acquire secretory or ciliated differentiation, and allow the study of cell lineage relationships and the regenerative ability of epithelial cells within the endometrium.^43^^,^^47^^,^^48^

EEOs have also been used for endometrial cancer characterisation and drug-response studies.^49^^,^^50^ In such studies, EEOs were derived from pathological endometrium, including from uterine pathologies that present with AUB i.e., hyperplasia and carcinoma (AUB-M). These EEOs accurately reproduce endometrial cancer subtypes, mutational landscape, and demonstrate patient-specific treatment responses *in vitro*.^51^^,^^52^ The establishment of such pathological models allows direct comparison between malignant-derived organoids and normal tissue-derived organoids from the same patient, while eliminating the “noise” from the patient's biological background.^51^

Endometrial epithelial organoids have also advanced our understanding of the pathogenesis of adenomyosis, which commonly presents with the symptom of AUB (AUB-A). For example, disruption of cell polarity, the asymmetric organisation of cellular components, has been hypothesised as a key mechanism of adenomyosis pathogenesis.^53^ With the use of the EEO model, it was found that oestrogen could disrupt the apical-basal polarity of epithelial cells, which may lead to myometrial cell invasion and establishment of an adenomyosis lesion.^53^

Like other cell culture models, EEOs have limitations. Firstly, the total cost of cell culture is increased due to increased experimental time for epithelial cells to self-organise and grow into organoids, and the multiple medium reagents that are required. Secondly, this model only assesses epithelial cell-specific function and does not consider the influence that stromal or immune cells may have upon epithelial cell function. Next, embedding cells in extracellular matrix naturally favours the organoids to form with the apical side, carrying the microvilli and cilia, facing towards the lumen of the organoid, limiting the accessibility of cell secretions. The extracellular matrix is also a limiting factor, as the most widely used Matrigel® is not chemically defined, has batch-to-batch variability, and may itself interfere with epithelial cell function. Lastly, the model lacks automation which could be useful for controlled and gradual administration of hormones to mimic the ever-changing *in vivo* endometrial environment.

Recent advances in developing 3D cell culture methods have demonstrated the importance of the nature of the scaffold used for this *in vitro* model of endometrial epithelial glands. Natural and semi-natural hydrogels have gained strong interest as alternatives to Matrigel® for epithelial organoids and other endometrial cell populations. For example, both fibrin and collagen, of which gelatine is a derivative, are proteins that regulate angiogenesis, making these gels suitable for *in vitro* studies on endothelial cell function and neovessel formation.^54^^,^^55^ Increased angiogenesis has been identified in patients suffering from adenomyosis.^56^ In this context, the fibrin and collagen-derived hydrogels can be applied to studies that investigate the exact role of angiogenesis in the aetiology of adenomyosis and in relation to AUB. While these natural biomaterials are well-suited for some studies, they may have batch-to-batch variability; their stiffness and degradation kinetics are not tuneable; may present immunologic reaction in studies involving more complex culturing system; and can only be culture short-term.^55^^,^^57^

Cook et al have described a well-defined, synthetic hydrogel, based on polyethylene glycol (PEG) that provides reliability and reproducibility of experimental analysis comparable to the widely used Matrigel®. This PEG hydrogel is supplemented with specific peptides that allow for dynamic response to endometrial cell secretions and cell-specific local ECM modulation.^58^ By systematically varying the components of this hydrogel, Hernandez-Gordillo et al recognised that the incorporation of the collagen-derived peptide GFOGER contributes to the robust success of epithelial organoid growth and reproducibility of results, demonstrating the advantage of working with a chemically defined hydrogel. Hernandez-Gordillo et al also showed the applicability of this synthetic ECM to wider fields, including the generation of intestinal and endometrial epithelial organoids.^59^ Recently, Gnecco et al generated an endometrial stromal cell and epithelial organoid co-culture model in this PEG-based hydrogel [pre-print].^60^ By dissecting the molecular and phenotypic consequences of the cross talk between cells, the authors demonstrated that this gel can sustain the dynamic changes of the endometrial microenvironment and can be applied when studying uterine health and disease [pre-print].^60^

### Endometrium-like stromal-epithelial co-culture models

The interdependence of stromal-epithelial interaction on endometrial function has been studied extensively through a 3D co-culture model, where both stromal and epithelial cells are embedded in a 3D matrix (Figure 4c). *In vivo*, aberration of intercellular communication may lead to endometrial dysregulation and the clinical complaint of AUB. Co-culture models are an essential tool for the investigation of cell communication. Key co-culture findings include the stromal oestrogen receptor mediation of epithelial mitogenesis,^61^ and the importance of stromal-epithelial crosstalk for epithelial secretion of metalloproteinases, which are essential for extracellular matrix remodelling during endometrial breakdown and regeneration.^62^ In addition, stromal-secreted decidualisation markers, for example, prolactin and IGFBP-1, also stimulate epithelial gland differentiation and secretion as demonstrated by co-culture systems.^63^

With the establishment of co-culture models, there has been an increasing interest in modelling gynaecological pathologies, such as adenomyosis, that presents with the symptom of AUB. Adenomyosis lesions have been difficult to model in a 2D environment due to their complex pathological processes and biological components. Co-culturing of endometrial stromal, epithelial, immune, and endothelial cells in combination with myometrial smooth muscle cells have enabled the generation of such a sophisticated model of adenomyosis lesion microenvironment *in vitro.* The prospects for applying such physiomimetic modelling to adenomyosis has been extensively reviewed by Gnecco et al in 2020.^64^

Co-culturing of endometrial cells can be a useful model to study cell-cell interactions *in vitro,* which could be perturbed in patients suffering with AUB*.* However, like other models, co-cultures can have key limitations that must be considered. Like the popularity of using Matrigel® for growing epithelial organoids, Matrigel® is also widely used for stromal-epithelial co-cultures. It has been previously reported that stromal cells embedded in Matrigel® lose their proliferative capability compared to stromal cells grown on plastic as a monolayer.^65^ In addition, the inversed cell polarity of epithelial organoids means there is limited access to cell secretions, as well as inability to generate a model of the lumenal portion of the endometrium to investigate cell function*.* Establishment of lumenal-only epithelium and stromal cell co-culture has been demonstrated using porous collagen scaffold. While this collagen-based scaffold preserved stromal and epithelial cell responsiveness to hormones, Abbas et al recognise that directing epithelial cells to form gland-like extension into the gel is still challenging.^66^ Further research efforts into the establishment of an improved and more physiological scaffold for stromal-epithelial co-cultures have led to the generation of a porcine-derived endometrial extracellular matrix (EndoECM).^67^ EndoECM is described to support stromal and epithelial cell co-culture, cell viability, ECM remodelling, and to improve stromal cell proliferation compared to collagen-based and Matrigel® co-cultures.^67^

Finally, tissue-derived organoids from kidney, stomach, and intestine have been used as a co-culture model in immunology studies of epithelial-immune cell interaction in tissue development, and has the prospect for applications in endometrial-specific research.^68^

### Endometrial organ-on-a-chip system

A step towards more “*near-to-in vivo*” studies is the establishment of an organ-on-a-chip model based on a microfluidic system. A microfluidic chip is a polymer device with moulded or engraved micro channels and chambers, through which fluids are injected, directed, mixed, or manipulated before evacuating the chip. Different designs of microfluidic chips exist, presenting various possibilities for experimentation with cells, organoids, and/or co-cultures.

A microfluidic model of the female reproductive tract was described in 2017 revealing a powerful tool to study hormonal signalling as a phenocopy of the menstrual cycle.^69^ In addition, Gnecco et al designed a microfluidic model of endometrial perivascular stromal cells and observed cellular response to changing hormonal stimulation. After simulation of laminar perfusion and haemodynamic forces, typical of endometrial blood flow, they found increased endothelial cell secretion of prostanoids and enhanced perivascular decidualisation of stromal cells.^70^

Microfluidic models offer an unprecedented opportunity to study endometrial physiology that is not possible with other pre-clinical models. They are permeable to gas, and accommodate dynamic flow of media and hormonal gradients at a microscale. This allows for the use of minimal volume of cells, reagents, and chemicals, making the system cheaper than conventional cell culturing models. In addition, they can be automated, where minimal manual handling is applied, reducing the risks of human error. The users may also have high experimental control by setting multiple mechanical, optical, chemical, and electrical biosensors to simultaneously measure cell and tissue function in real-time. They offer faster reaction time, enhanced analytical sensitivity, portability, and do not require costly equipment.

This experimental model also presents some challenges. Re-locating cells from standard cell culture dishes to microfluidic chips require revision of culture protocols to account for the reduced medium volume and different polymer surfaces. There is growing evidence for differing cell responses between macro and micro cultures in consideration of change in growth surface physical properties, cell numbers, volume densities, nutrient consumption, and pH regulation.^71^ Next, the effect of structural abnormalities such as the presence of large uterine fibroids *in vivo*, cannot be studied using this model. Finally, the lack of adipose cells to absorb lipophilic chemicals may also alter the results in drug discovery experiments, which is particularly important when studying drug distribution. Similarly, the most widely used silicon-based polydimethylsiloxane (PDMS) to construct microfluidic devices can release uncured PDMS into the culture medium and can absorb lipophilic molecules, which alters the experimental results.^72^ Emerging technologies are addressing such fundamental issues and novel microfluidic platforms that become available have overcome some of these limitations.^73^

## Human *ex vivo* model

### Organotypic *ex vivo* models to study AUB and uterine fibroids (leiomyoma)

Another approach for studying AUB is the use of an *ex vivo* model, usually thinly cut whole tissue slices, mounted onto a porous membrane, and cultured under controlled conditions. This “*in vivo*-like” model, preserves the natural architecture, cellular diversity and networks, cell viability, systemic gene and protein expression patterns, and pathway activity of the tissue of origin.^74^ Often, this technique is employed for studying tumours of lung, prostate, gut, and breast, however, recent advancement also shows establishment of uterine fibroid (leiomyoma) organotypic cultures.^75^ Salas et al describe uterine leiomyoma slices cultured on an alginate scaffold that respond to hormonal stimuli and express genes associated with leiomyoma development. The alginate scaffold has been constructed to contain microspheres with encapsulated drug, which offers a novel approach to investigate patient-specific drug responses.^75^

The limitation of this model is accessibility to human-derived whole tissue biopsy and short culture maintenance of up to five days.^74^ Despise this, the potential of organotypic *ex vivo* constructs as a personalised pre-clinical model for cytotoxicity studies to targeted therapies is widely recognised.

## Current pre-clinical models to study non-human endometrium and AUB

### Non-human *in vivo* models of menstruation

The regulation and mechanism of menstruation is ideally studied in humans. However, human heterogeneity and the inevitable disruption of endometrial architecture during tissue sampling may limit findings. Animal models of menstruation provide genetic homogeneity and allow genetic, environmental, and pharmacological manipulation to determine causation.

### Non-human primates

Non-human primates menstruate and undergo spontaneous endometrial decidualisation. Rhesus macaques have similar uterine morphology and length of menstrual cycle to humans.^76^ Both species display tightly co-ordinated spatial and temporal regulation of endometrial physiology at menstruation, e.g., increased MMPs and VEGF, vasoconstriction of spiral arterioles, and local tissue hypoxia.^77^^,^^78^ These observations are preceded by progesterone withdrawal.^77^^,^^79^^,^^80^ Macaques are reported to experience menstrual abnormalities (e.g. heavy menstrual bleeding) and may be fitted with tampons, and are excellent candidates for evaluating therapies for menstrual disorders.^78^ Despite menstruating naturally, macaques may undergo ovariectomy and treatment with oestrogen and progesterone to create artificial menstrual cycles and accurate timing of endometrial sampling. Use of this model requires large experimental groups and long experimental times, resulting in significant costs. Therefore, many researchers now preferentially use rodent models to study menstruation.

### Mouse models of simulated menses

**Figure 5:**
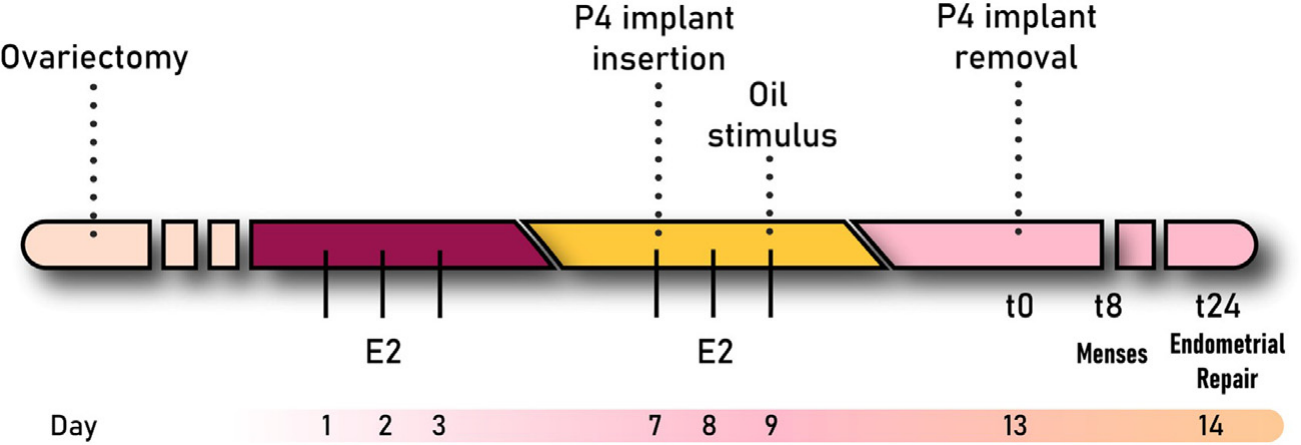
**Mouse model of simulated menses.** Ovariectomy is performed on female mice to deplete endogenous levels of ovarian hormones. After allowing 7 days for surgery recovery, mice are given daily subcutaneous injections of oestradiol (E_2_) for three days (days 1-3). Seven days after the first E_2_ injection, a progesterone (P_4_) implant is subcutaneously inserted (day 7) along with a lower dose of E_2_ (days 7-9). In order to trigger decidualisation, oil is transcervically administered at day 9. The removal of the P4 implant (t0) recapitulates menstrual events, with mice bleeding 8h after P_4_ withdrawal (t8). Twenty-four hours after P4 withdrawal (t24), endometrial regeneration events can be observed.^81^^,^^82^

The mouse model of simulated menstruation was initially described in 1984^81^ and further optimised in the 2000’s^82^ (Figure 5). Mice are ovariectomised and supplemented with exogenous oestrogen and progesterone to mimic the human hormonal endometrial environment. The model requires artificial induction of decidualisation, via a transcervical or surgical intrauterine injection of oil, reinforcing the importance of a decidualisation step prior to progesterone withdrawal in the physiology of menstruation. Once decidualisation has taken place, progesterone withdrawal results in histological and molecular changes analogous to those observed in the human endometrium at menstruation, with recruitment of leukocytes,^83^ shedding of the luminal endometrium, and visible menstrual-like bleeding.^84^ Endometrial tissue is then repaired and remodelled. Alternatively, simulation of menses may be achieved by inducing pseudopregnancy. In this model, female mice are mated with vasectomised males to mimic fertilisation events. Progesterone withdrawal may occur naturally, by ovariectomy, or by administration of a progesterone antagonist.^85^

Despite endocrine (e.g., a shorter length of cycle and lack of spontaneous decidualisation) and immunological differences between mice and humans,^86^ exogenous hormonal supplementation permits recapitulation of the events of human menstruation and decidualisation in this murine model.^5^^,^^87^^,^^88^ Consistent with findings in the human endometrium,^89^ the mouse model also has a critical period of progesterone withdrawal, where replacement of progesterone early after its withdrawal prevented menstrual-like bleeding and endometrial shedding. However, progesterone replacement at later time-points did not suppress menstruation.^90^ It is also a popular model to investigate the dynamics of endometrial breakdown and repair and has been used to manipulate inflammation^91^ and the endocrine environment^92^ at menstruation, and to pharmacologically and genetically alter key factors involved at menses, for example, hypoxia^93^^,^^94^

### Xenograft mouse model

The xenograft mouse model provides an alternative model for *in vivo* examination of menstrual physiology and pathology.^95^, ^96^, ^97^ In this model, fragments of human functional endometrium are xenografted to ovariectomised, immune-deficient mice, most commonly the severe combined immunodeficiency (SCID) mouse. Treatment with oestrogen and progesterone followed by removal of ovarian steroids resulted in menstrual breakdown of the xenografted human endometrium. Xenograft menstruation studies have examined endometrial regeneration and the role of ovarian steroids in regulating this process.^95^^,^^98^ The advantages of this model are the ability to standardise ovarian hormone variations, manipulate the endometrium in a way that would be unethical in humans, and examine the local versus systemic leukocyte response by identification of human and mouse cell contributions. Limitations of the model include significant compromise of endometrial tissue architecture following transplantation, which may alter vascular and cellular responses. In addition, the necessary immunosuppressed state of the recipient mice may alter physiological inflammatory events in the menstrual endometrium. However, the commonly used SCID model aims to suppress T and B-cell mediated xenograft rejection without substantially affecting the innate immune response, and may be more relevant than other immunocompromised recipient mice.^95^

### Spiny mouse

The common spiny mouse (*Acomys cahirinus*) is the only known rodent to display spontaneous decidualisation and natural menstruation, with menstrual duration of 3 days and frequency of 6-10 days.^99^^,^^100^ This rodent has previously been studied as an animal model for obesity and diabetes mellitus, and therefore, some laboratory reagents are currently available. The spiny mouse uterus is anatomically different to the human, but has physiological similarities, such as spiral arteriole remodelling in the perimenstrual phase.^99^ In addition, endometrial decidualisation is tightly controlled, not compromising the structural integrity of the endometrial glands or the myometrium, as observed in other rodent models. Like women, the spiny mouse produces cortisol as its circulating glucocorticoid, as opposed to corticosterone in standard laboratory mice. This rodent may permit study of multiple successive menstrual cycles and examination of any pre-conditioning effects that menstrual cycles will have on endometrial physiology. Disadvantages include the variability of natural cycles, the current inability to genetically manipulate the model, limiting definitive mechanistic studies, and lack of specific antibodies and molecular biological reagents (e.g., primers).

### Non-human *in vivo* models of AUB

#### Adenomyosis models

Adenomyosis occurs spontaneously in different mammals, including non-human primates,^101^ mice, rats and rabbits. While these animal reports have proven to be useful for studying the development of adenomyosis, timing of onset and variability in the numbers affected limit use as a pre-clinical model for therapeutic intervention. In the 1980s, hormonal carcinogenic studies on mice revealed that exposure to certain endocrine-disrupting chemicals increased the risk of developing adenomyosis, alongside other uterine abnormalities such as cervical abnormalities, mammary carcinomas^102^ and endometriosis.^103^ Pre-natal oral exposure to diethylstilbestrol (DES),^102^ dioxins^104^ or phthalates^105^ generated adenomyosis. However, the incidence is strain-dependent, with DES-exposed CD-1 mice being protected from developing adenomyosis, but prone to uterine malignant tumours,^106^ and BALB/c mice showing complete protection from any uterine abnormalities.^102^ Post-natal DES administration also displayed varying results depending on administration route. For example, using the same mouse strain, intraperitoneal administration of DES generated adenomyosis,^107^ while subcutaneous administration showed development of uterine carcinomas.^108^ Therefore, induction of adenomyosis using endocrine-disrupting chemicals is limited by lack of specificity and a long induction time (6 to 20 months after treatment) that introduces variability with age.

One interesting finding in the DES model was the increased incidence of hyperprolactinemia observed in mice developing adenomyosis.^102^ This observation triggered the development of adenomyosis models relying on hyperprolactinemia replication via implantation of pituitary grafts from other mice or pharmacological induction with dopamine agonists, or selective serotonin reuptake inhibitors.^109^^,^^110^ Interestingly, the number of pituitary glands implanted, and the donor-recipient match/mismatch did not influence adenomyosis incidence.^109^ However, the site of implantation did affect incidence, with transvaginal^111^ or surgical uterine lumen implantation^112^ displaying increased adenomyosis rates than kidney capsule implantation.^113^^,^^114^ As in previous models, adenomyosis incidence was strongly strain-dependent, with BALB/c mice being protected compared to other strains.^115^ In contrast to the endocrine-disrupting chemical models, this pituitary grafting model displays a shorter endpoint (∼3 months) and has also been validated in rats.^116^ This model has contributed to research on novel therapeutics^117^^,^^118^ and the study of molecular aspects of adenomyosis.^113^^,^^119^ Limitations include the significant variability in adenomyosis induction rates, even within the same research group.^114^^,^^120^ In addition, specific induction of adenomyosis remains elusive, with co-development of other uterine abnormalities.^112^

The most popular current model of adenomyosis is the tamoxifen model.^121^ Oral administration of this selective oestrogen receptor modulator from day 2 to 5 after birth results in an adenomyosis induction rate of 100%.^121^ One of the reasons for its success is the short induction time of the lesions: at days 5-10 there is early evidence of adenomyosis^121^ and by day 42 from birth, the model is completely established.^122^ Despite its time advantage, the tamoxifen model presents similar limitations to the previous models, displaying strain and route of administration dependency with C57/BL6J mice protected^123^, and subcutaneous injection resulting in uterine carcinoma development.^123^

Very recently, Hao et al. have developed two mouse models of adenomyosis by disrupting the interface between the endometrium and the myometrium.^123^ This disruption is successfully achieved by either a mechanical or a thermal stimulus. With mechanical induction, a microcatheter is inserted into a uterine horn and the tip used to damage the endometrial-myometrial interface. For thermal induction, the microcatheter is attached to an electrosurgical scalpel, generating disruption by electrocoagulation. Although technically more complex, this model seems to be strain independent and provides an opportunity to adjust the severity of the lesions by increasing the damage generated**.**^123^

#### Uterine fibroid (Leiomyoma) models

Uterine fibroid (leiomyoma) formation in rodents occurs following exposure to endocrine-disrupting chemicals.^124^ Rat neonatal exposure to DES, bisphenol A,^125^ or tributyltin^126^ promotes the development of leiomyoma, as well as other uterine abnormalities such as glandular hyperplasia^127^ or adenomyosis.^107^ Additionally, some mouse strains also report leiomyoma incidence when prenatally or neonatally exposed to some of these compounds.^128^ Despite the non-specificity of their phenotype, these models are still in use for the exploration of potential treatments against leiomyoma formation and development.^129^ However, genetic models such as the Eker rat model have gained popularity.

Eker rats, which are defective for the tumour suppressor gene Tsc2, spontaneously develop uterine fibroids in approximately 65% of cases.^130^ Alterations in this gene are also observed in some women with leiomyoma.^131^ The course and composition of the lesions in rats are similar but not identical to human lesions.^132^ Eker rats have proven useful to study the hormonal influence on the development of leiomyomas.^133^ In mice, the Tsc2 mutation phenotype is not replicated but mutations in the Med12 gene do generate leiomyomas.^134^ This gene is affected in up to 80% of women with leiomyoma,^135^ offering another potential model of genetic predisposition. The most common critique for both these genetic models is that a single mutation may oversimplify this highly complex uterine condition. As a refinement option, the Eker rat model can be combined with endocrine-disrupting chemicals. Neonatal exposure to DES in Eker rats significantly increased leiomyoma formation from 65% to a 100%.^136^ This combined model has been used to study the molecular triggers of uterine leiomyoma development as well as potential treatments.^137^

The mouse xenograft model is an established pre-clinical model for developing novel therapeutics to suppress leiomyoma growth.^138^^,^^139^ In the most conventional model, leiomyoma tissue derived from patients is cut in sections or disaggregated and further implanted on different immunosuppressed mice strains. The preferred graft location tends to be subcutaneous^140^, although some reports suggest that sub-renal implantation displays better engraftment.^141^ Intrauterine transplantation has also been tested but displays less leiomyoma formation than other routes.^142^ Tissue sections and freshly isolated cells derived from tissue disaggregation display similar reproducibility and level of engraftment.^143^ Selection of recipient mice remains controversial, with some studies claiming that the optimal engraftment is achieved using SCID mice,^132^ and other reports favour lymphocyte T, B, and NK cells-deficient mice (NOD/SCID mice).^141^ The xenograft model has also been tested on rats where, rather than generating a genetic immunodeficiency, animals are treated with mycophenolate mofetil to avoid transplant rejection.^144^ In every model, hormonal supplementation is essential for graft survival, with administration of both oestrogen and progesterone displaying the best results.^143^ This xenograft model has been used to transfect tissue (or cells) with plasmids,^145^ as well as microRNAs to study the impact on their development.^146^ Very recently, a new xenograft model was validated, where human leiomyoma cell lines were 3D-cultured prior to subcutaneous implantation in immunosuppressed mice.^147^ This removes the requirement for fresh tissue samples, reduces donor intra-variability, and showed longer graft survival (over 8 weeks).^132^

## Outstanding questions

The evolution of *in vitro* models of endometrial-derived, multi-cellular, and three-dimensional constructs have shown their potential for uncovering the function of individual cell types, inter-cellular interaction, and cell interaction with its microenvironment. The advancement of the complexity of these cultures has served as a proof-of-concept that establishment of an *in vivo*-like endometrial microenvironment is possible. Forward-looking, modern gene and protein discovery techniques can now begin to describe in a non-biased manner the key cellular and molecular responses of the uterus that result in structural and non-structural causes of AUB. Non-structural causes such as AUB-E require delineation of mechanism of action for treatment improvement, whereas structural causes such as uterine fibroids require delineation of endometrial impact as a cause (or lack) of fibroid-associated AUB and *vice versa*. Gaining such understanding can be possible with the emerging *in vitro* models, which can help address the complexity of the presence of more than one cause of AUB in a single patient. In addition, *in vivo* models are still necessary to study the organ-to-organ interactions which may equally impact normal and abnormal uterine function.

## Concluding remarks

Pre-clinical models have significantly advanced our understanding of endometrial function and AUB. While every pre-clinical model offers research advantages, each has specific limitations as discussed. For this reason, careful consideration and selection criteria are required when choosing the right model system or combination of models for a specific research question. Correct research approaches can answer scientific questions, lead to therapeutic target identification/validation and inform clinical trial design. It is an exciting time for research in the reproductive field and recent advancements have the potential to identify new approaches to ameliorate abnormal uterine bleeding (AUB).

## Search strategy and selection criteria

Data for this Review were identified by searches of the National Institute for Health and Care Excellence website, PubMed, Scopus, ScienceDirect and references from relevant articles using search terms “abnormal uterine bleeding”, “endometrial stromal/epithelial cell cultures”, “endometrial organoids”, “endometrial co-cultures”, “microfluidic”, “adenomyosis”, “leiomyoma “, and “models, animals”. Boolean operator “AND” and truncation strategies were used. Only articles published in English were included with focus on publications between 2015 and 2022. There are several exclusions where description of model development is discussed, and publication year precedes this period.

## Contributions

All authors made significant contributions to manuscript preparation and read and approved the final versions and revisions of the manuscript.

Aleksandra O Tsolova: literature search, conceptualisation, figures, writing – original draft, and writing – review & editing.

Rocío Martínez Aguilar: literature search, figures, writing – original draft, and writing – review & editing.

Jacqueline A Maybin: writing – original draft, and writing – review & editing.

Hilary OD Critchley: conceptualisation, original draft; writing — review & editing.

## Funding

Hilary OD Critchley receives support from the Medical Research Council Centre for Reproductive Health (MRC CRH) Grant MR/N022556/1; and from Biotechnology and Biological Sciences Research Council (BBSRC; BB/S002995/1)

Aleksandra O Tsolova receives support from the Medical Research Council Centre for Reproductive Health (MRC CRH) Grant MR/N022556/1 and student grant MR/P502030/1

Jacqueline A Maybin receives support from Wellcome Trust Fellowship 209589/Z/17/Z and from the Royal Society of Edinburgh 1077.

Rocío Martínez Aguilar receives salary support from Wellcome Trust Grant 209589/Z/17/Z.

The funders had no role in manuscript design, data collection, data analysis, interpretation, writing of this manuscript.

## Declaration of interests

Hilary Critchley (HC) has received clinical research support for laboratory consumables and staff from Bayer AG and provides consultancy advice (paid to Institution) for Bayer AG, Gedeon Richter, Vifor Pharma UK Ltd,; Myovant Sciences GmbH. HC has received royalties from UpToDate for article on abnormal uterine bleeding.

Jacqueline A Maybin receives Wellcome Trust Clinical Research Career Development Fellowship (salary support) - To institution; Tenovus Scotland - To institution; Royal Society of Edinburgh - To institution; Wellbeing of Women - To institution.
